# Shifting to virtual breastfeeding counseling for low-income women in the US during COVID-19: A partner-engaged multimethod evaluation of program adaptations

**DOI:** 10.3389/frhs.2022.1020326

**Published:** 2022-11-16

**Authors:** Elizabeth C. Rhodes, Helen Wilde LaPlant, Mahrukh Zahid, Nafeesa Abuwala, Grace Damio, Carrianne Crummett, Rebecca Surprenant, Rafael Pérez-Escamilla

**Affiliations:** ^1^Yale School of Public Health, Yale University, New Haven, CT, United States; ^2^Hispanic Health Council, Hartford, CT, United States; ^3^Trinity Health Of New England, Hartford, CT, United States

**Keywords:** adaptation, implementation, evaluation, health equity, community health worker (CHW), qualitative research

## Abstract

**Background:**

The Breastfeeding Heritage and Pride program (BHP) provides evidence-based breastfeeding peer counseling to low-income women. Due to the COVID-19 pandemic, BHP shifted from delivering in-person and virtual services to providing only virtual services. Program adaptations can impact implementation success, which could influence program effectiveness. We documented program adaptations and explored their impacts on implementation outcomes, guided by the Model for Adaptation Design and Impact.

**Methods:**

Through a community-clinical-academic partnership, we conducted in-depth interviews with 12 program implementers and peer counselors and conducted a rapid qualitative analysis. To efficiently capture information on adaptations over time, we collected and analyzed information from program meetings and extracted data from a program report. We then triangulated data from these multiple sources.

**Results:**

Peer counselors received training on virtual service delivery and increased supportive supervision. They recruited women via phone instead of in hospitals, which was viewed as feasible. In-person counseling visits at hospitals and clients' homes were replaced with phone and video calls. Examples of changes to the content delivered included breastfeeding education in the context of the pandemic such as the latest COVID-related infant feeding guidance, provision of face masks, and more assistance with social and economic challenges. Although peer counselors increasingly adopted video calls as a substitute for in-person visits, they emphasized that in-person visits were better for relationship building, helping with breastfeeding problems like latching, and identifying barriers to breastfeeding in the home environment like limited familial support. While adaptations were reactive in that they were made in response to the unanticipated COVID-19 pandemic, most were made with clear goals and reasons such as to ensure the safety of peer counselors and clients while maintaining service delivery. Most adaptations were made through a systematic process based on program implementers' expertise and best practices for peer counseling and were largely but not fully consistent with BHP's core functions.

**Discussion:**

BHP was able to shift to virtual service delivery for continued provision of breastfeeding counseling during the pandemic. Overall, virtual services worked well but were less optimal for several aspects of counseling. Evaluations of program effectiveness of virtual services are still needed.

## Introduction

Community health workers (CHWs) are an expanding part of the healthcare workforce in the United States (1). CHWs play a pivotal role in advancing healthcare equity by increasing access to and supplementing a range of health services for low income and other underserved populations ranging from chronic disease prevention and management to mental health and maternal-child health care (2–5). CHWs are trained community members who often share cultural backgrounds and lived experiences with clients, enabling them to build trusting relationships with clients and serve as a link between clinical and community-based health services (6). CHW programs improve health outcomes, reduce health inequities, and produce cost savings from lower healthcare utilization (3, 4, 6–8). Many programs provide services in community settings like community-based organizations, clients' homes, and some also in hospitals (6, 9). The COVID-19 pandemic precipitated a shift to virtual delivery of CHW programs to lower risk of infection for both CHWs and clients, while maintaining access to services at a time when healthcare and health inequities were exacerbated (10). Program implementers are now drawing on the innovative and promising practices employed during the pandemic to inform future program changes, such as permanently integrating telehealth to lower program implementation costs and increase the accessibility and scalability of services. Despite the ever-increasing use of telehealth, the changes made to CHW programs to deliver virtual services, as well the impact of these changes on implementation success, have not been well evaluated.

Previous research indicates that digital health tools such as telehealth need to be tailored to the context. Socio-economically disadvantaged individuals and communities are more likely than better-off groups to have less access to digital technology as well as lower digital literacy which has been linked to discomfort with telehealth or low uptake (11). The COVID-19 pandemic may have prompted changes to the content of health education delivered by CHWs, such as shortening education, breaking up content over multiple sessions, or adding elements related to SARS-CoV-2. These changes can influence implementation success in both intended and unintended ways (12). For example, replacing in-person home visits with video calls could increase acceptability of face-to-face interactions when CHWs want to minimize exposure to SARS-CoV-2 but reduce appropriateness if services require hands-on support. Understanding and optimizing implementation success is critical, since an intervention will not be effective if implementation is poor (13). Implementation outcomes such as feasibility, appropriateness, acceptability, and adoption serve as indicators of the success of implementation (13). Systematically documenting changes to CHW programs, as well as their impact on implementation outcomes, could enable program implementers to understand how to make changes that optimize implementation success across different target populations and settings while avoiding changes with potential negative effects on implementation that can in turn compromise effectiveness (14).

This study was conducted by researchers through an equitable partnership with community and clinical partners implementing the Breastfeeding Heritage and Pride program (BHP), an evidence-based breastfeeding peer counseling program for women with low incomes delivered by specialized CHWs known as peer counselors (9). In response to the COVID-19 pandemic, BHP temporarily shifted from providing in-person and virtual services to only delivering virtual services. Our objectives were to systematically document changes to the program and explore their impacts on implementation outcomes. The findings offer program implementers timely and practical information that can facilitate proactive decisions about changes that can enhance telehealth implementation within the context of breastfeeding peer counseling programs, as well as other CHW programs.

## Materials and methods

### Breastfeeding Heritage and Pride program

**Figure 1:**
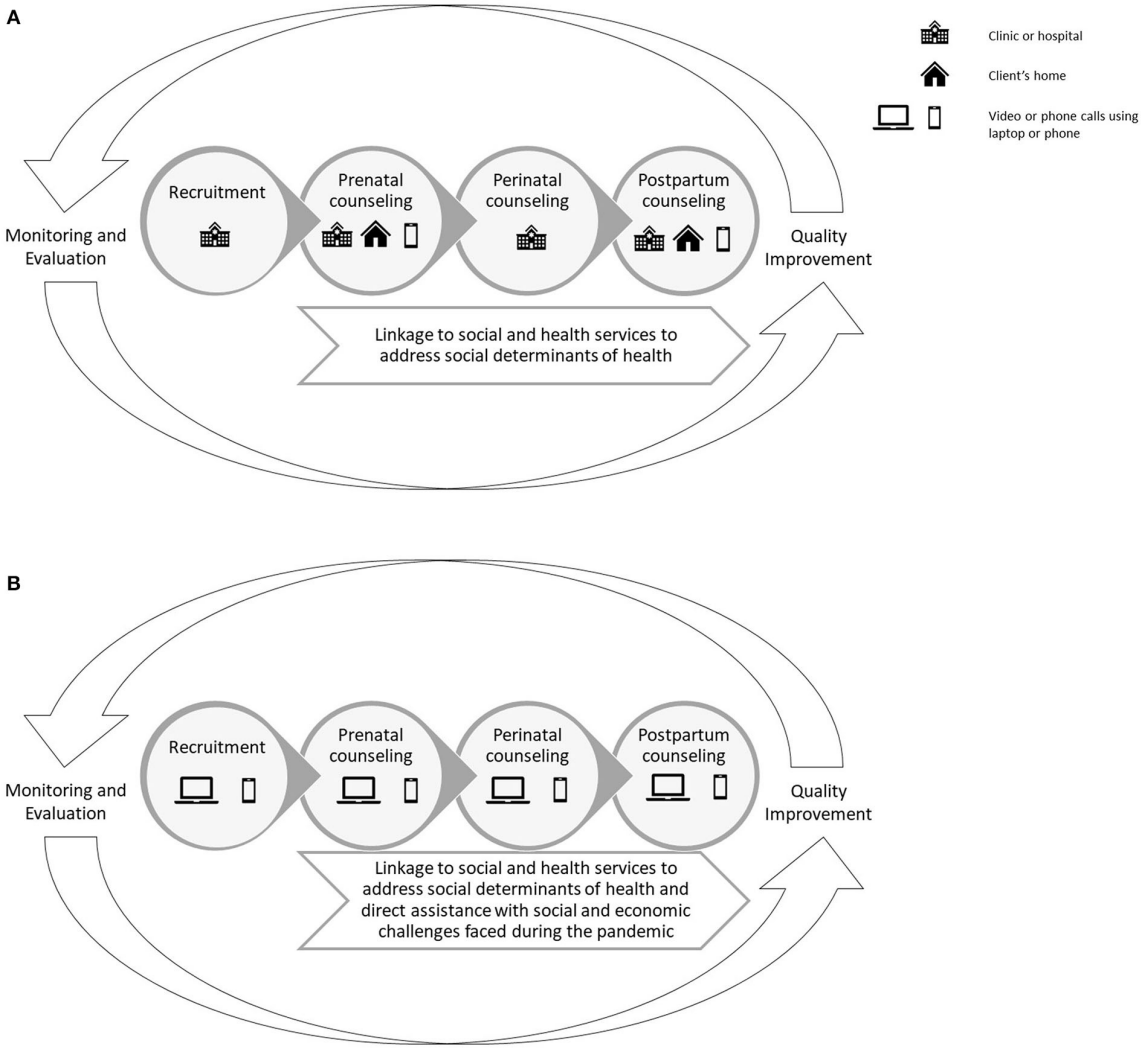
**(A)** Pre-pandemic continuum of breastfeeding support delivered by the Breastfeeding Heritage and Pride program. The Breastfeeding Heritage and Pride program (BHP) offers a continuum of breastfeeding counseling to clients across the prenatal, perinatal, and postpartum periods. Peer counselors provide in-person breastfeeding education and hands-on lactation management support in healthcare facilities and clients' homes, as well as breastfeeding information and support via video calls, phone calls, and text messages. BHP connects clients with a range of social and health services able to assist with addressing social determinants of health that can make breastfeeding difficult or adversely impact the health and well-being of clients and their families. A key component of BHP is the monitoring and evaluation system designed to facilitate continuous quality improvement of breastfeeding peer counseling services. Figure developed by Rhodes and colleagues and originally published by BMC (9). **(B)** Continuum of breastfeeding support delivered by the Breastfeeding Heritage and Pride program during the COVID-19 pandemic. The Breastfeeding Heritage and Pride program (BHP) continued to offer the same continuum of breastfeeding support across time but shifted to using primarily phone or video calls in place of in-person visits in healthcare facilities and clients' homes. BHP expanded support for clients with social and economic needs by offering direct assistance.

BHP was created in 1993 by the Hispanic Health Council (HHC), a community-based organization promoting health equity. Detailed descriptions of the community-engaged process for designing and implementing BHP, as well as the rigorous program evaluations and their findings, have been presented elsewhere (9). Figure 1 depicts the continuum of breastfeeding support delivered by BHP. Briefly, HHC hires women who have breastfed successfully for at least 6 months and trains them to be peer counselors through an evidence-based curriculum and practice-based mentored learning. Peer counselors can share strategies for overcoming breastfeeding challenges based on their personal breastfeeding experiences and their training, enabling them to build trust as peers and serve as role models. HHC partners with healthcare systems serving predominately women of color with low incomes to deliver BHP. Peer counselors are integrated into prenatal and postpartum clinical care teams and based at hospitals. Healthcare providers refer pregnant women to BHP, who are then contacted and recruited into the program by peer counselors. Additionally, peer counselors use Epic, an electronic medical record system, to identify women scheduled for prenatal visits. Once identified, peer counselors meet women when they come to prenatal clinics for their visits, introduce them to BHP, and offer to enroll them in the program.

Women who enroll in the program receive free breastfeeding education and lactation management support from peer counselors through one-on-one in-person counseling sessions, which are held in partner healthcare facilities, women's homes, and other community settings. These sessions begin prenatally and continue up to one year postpartum and are guided by a protocol that specifies the key topics for peer counselors to cover during each visit, as well as the number, timing, and location of visits. Clients are offered at least: three prenatal visits, which are typically delivered in prenatal clinics; one in-hospital perinatal visit soon after delivery; and five postpartum home visits, supplemented by seven phone calls. A client who wants or needs more support receives additional in-person visits and phone calls. The protocol was designed so that services are delivered in locations that make services highly accessible. Visits are planned in locations that are most convenient for clients and best enable peer counselors to address their needs. Prenatally, in-person contact facilitates building rapport and addressing barriers to deciding to breastfeed that are sometimes emotional, cultural, and/or socio-economic. Postpartum in-person contact allows for hands-on lactation management support. It is also important for observation of the home environment to identify factors that may enhance or diminish breastfeeding success, such as the degree of breastfeeding support in the home and socio-economic circumstances.

Bilingual peer counselors communicate with clients in English or Spanish according to each client's preferred language, and use electronic translation services for clients that speak other languages. To help retain clients in the program, peer counselors conduct active outreach through phone, email, text message, mail, and home visits. To promote high-quality counseling, BHP International Board Certified Lactation Consultants (IBCLCs) provide peer counselors with ongoing training and clinical guidance through regular group and individual meetings, onsite observation, and phone calls as needed. Program IBCLCs also directly support clients who face breastfeeding challenges that require more specialized knowledge and skills. A program manager provides peer counselors with supportive supervision tailored to their specific supervision needs. When clients face housing and food insecurity or other social determinants of health challenges, they are connected by the program manager to health and social services to address them, as this is outside the scope of the specialized peer counselor role.

Continuous quality improvement is supported by a robust monitoring and evaluation system. During each client contact, peer counselors collect data that are entered into a data management system. The data are used to generate assessments of process indicators (e.g., number of clients enrolled, number of contacts such as those for client outreach and counseling visits) and clients' breastfeeding goals and outcomes such as exclusive breastfeeding for 6 months, which informs the program manager as she supports peer counselors and promotes quality assurance.

Core functions of the program were identified through a process guided by the method described by Kirk and colleagues for identifying and reporting core functions and forms of evidence-based interventions *post-hoc* (15). Briefly, we conducted audio-recorded, semi-structured interviews with four program developers to elicit information about the needs and problems that motivated the development of BHP (i.e., motivating needs), core functions (i.e., core purposes of the evidence-based intervention that make it effective and, thus, should be preserved), and forms (i.e., specific activities that are needed to carry out the core functions and that may be adapted) (16). Next, we transcribed the data verbatim, coded transcripts, and analyzed the data to identify and map forms to the core functions they fulfill as well as to identify and map core functions to the motivating needs they address. To visually depict our findings, we created a BHP function and form matrix with three main columns: motivating needs, core functions, and forms (16). Finally, we iteratively refined the matrix through a series of group discussions with program developers and implementers. Detailed methods for identifying core functions will be described in a forthcoming paper.

The core functions of the program are three-fold. First, the program provides integrated peer counselor-delivered services across clinical and community settings. Second, the program provides high-quality breastfeeding education and lactation management services, with an emphasis on services that are accessible, evidence-informed, and person-centered. Third, the program promotes breastfeeding as a social norm and an empowering practice by hiring peer counselors who have successfully breastfed to serve as role models for clients.

Findings from two randomized controlled trials showed that BHP improves any and exclusive breastfeeding among women with low incomes (17, 18). BHP has since been endorsed as an evidence-based program by both the Centers for Disease Control and Prevention and National Academies of Science, Engineering and Medicine (19, 20). Most recently, the World Health Organization spotlighted BHP as an exemplary program in their report on implementation guidance on breastfeeding counseling (21).

### Study setting and partners

This study built upon a Kellogg-funded project to deliver BHP in three Trinity Health Of New England (Trinity Health) hospitals in Connecticut and Massachusetts. The project brought together a core implementation team comprised of BHP leaders from HHC (i.e., BHP founder, director, and manager), Trinity Health staff (i.e., physician, project replication manager, and assistant), and researchers from the Yale School of Public Health (RPE and ER), one of which (RPE) served as lead BHP evaluator for three decades. To optimize program implementation, they held semiannual meetings at each hospital with BHP peer counselors and providers supporting integration of BHP into the healthcare setting, such as physicians, nurses, medical assistants, IBCLCs, and midwives. The purpose of these meetings was to jointly apply a program impact pathway (PIP) method, which involves using a causal map of the steps and processes linking program goals, activities, and outcomes and identifying bottlenecks along the pathway, and then to devise ways to improve program implementation (9). When COVID-19 emerged, the first author (ER) led and worked collaboratively with the implementation team to conduct the present study.

### Conceptual model

The study as initially designed applied the Framework for Reporting Adaptations and Modifications-Enhanced (FRAME) by Stirman and colleagues to systematically document adaptations to BHP. It also applied the Implementation Outcomes Framework by Proctor and colleagues to evaluate the implementation of the adapted program, with a specific focus on three implementation outcomes: feasibility, appropriateness, and acceptability (13, 22). While data collection and analysis were underway, the Model for Adaptation Design and Impact (MADI) to promote systematic assessment of impacts of adaptations was published (12). MADI was developed by Kirk and colleagues by reviewing, consolidating, and refining constructs from the FRAME, Moore's research that proposes relationships between adaptation characteristics and outcomes, and the Implementation Outcomes Framework (12). We transitioned to using MADI to guide our data analysis and as a scaffolding for presenting the findings because it offered a way of combining the frameworks we had previously selected to guide our study, incorporating core functions, and moving toward an explanatory model (12).

MADI is comprised of three domains. Domain 1, adaptation characteristics, includes constructs from the FRAME: what was modified, the nature of the adaptation, who participated in decision-making, and for whom/what the adaptation was made. Based on Moore's research, Domain 2 consists of possible mediators or moderators of the impact of adaptation characteristics on outcomes, namely constructs from the FRAME. These constructs are whether the adaptation aligns with core functions of the intervention, reasons for the adaptation, whether the adaptation was systematic, and whether the adaptation was proactive. Domain 3 comprises implementation and intervention outcomes delineated in the Implementation Outcomes Framework that could be impacted by adaptations (12). MADI proposes that implementation and intervention outcomes may in turn affect impact of the intervention.

### Documentation and evaluation approach

**Figure 2:**
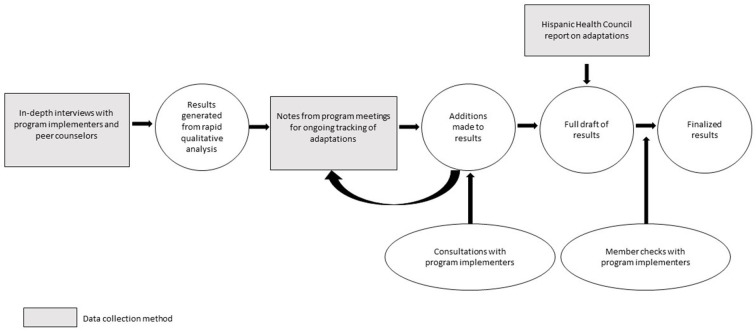
Process of documenting and evaluating adaptations to the Breastfeeding Heritage and Pride program made in response to the COVID-19 pandemic.

We used a qualitative multimethod approach to document and evaluate adaptations between April and December 2020 (Figure 2), which is described in detail below. In-depth interviews were conducted with program implementers and peer counselors, and the data were analyzed using a rapid analysis approach. Notes from bimonthly program meetings allowed for ongoing tracking of adaptations, and the information was added to the results from in-depth interviews in consultation with program implementers through an iterative process. Information from an HHC report on adaptations was extracted and incorporated into the full draft of results. Member checks with program implementers were completed to validate the results prior to finalization.

#### In-depth interviews

We conducted in-depth interviews with program implementers (i.e., one HHC senior leader and four members of the core implementation team from HHC and Trinity Health) and all seven peer counselors. We developed, pilot tested, and refined two semi-structured interview guides, one tailored for program implementers and one for peer counselors (see Supplementary materials 1, 2). These guides were informed by the FRAME to elicit information on adaptations and by Proctor's Implementation Outcomes Framework to elicit views on the influence of these adaptations on implementation success, with a focus on feasibility, appropriateness, and acceptability. Interviews with program implementers were conducted in English, and interviews with peer counselors were conducted in English or Spanish. Interviews were conducted between April and mid-June 2020 via Zoom, audio-recorded, and transcribed verbatim and translated into English if applicable. Peer counselors received gift cards for participation. To generate timely results for program implementers, we used a rapid analysis approach (23). We first created a summary of each interview transcript using a structured template and then transferred summaries into an Excel matrix. Next, we wrote detailed descriptions of reported program adaptations and their perceived impacts on implementation outcomes including adoption, feasibility, appropriateness, and acceptability.

Applying MADI, we categorized adaptations into three types: content (defined as changes made to the intervention procedures, materials, or delivery), contextual (defined as changes made to delivery of the same program content, but with modifications to the format or channel, the setting or location in which the overall intervention is delivered, or the personnel who deliver the intervention), and training and supportive supervision (defined as changes made to the procedures for training and providing supportive supervision to personnel) (24). We then created a table for each type of adaptations and populated the tables with the following elements as relevant: nature of adaptation; who participated in the decision to make the adaptation; for whom/what the adaptation was made; alignment with core functions; and the reasons for the adaptation, including the intent or goal of the adaptation and contextual factors that influenced the decision. We did not evaluate when adaptations occurred, an element included in MADI with four response options (pre-implementation, implementation, scale up, maintenance), since adaptations were made during implementation only. Coding categories were adapted from MADI to be applicable to this study (see Table 1).

**Table 1:** Methods for describing adaptation characteristics and possible mediating or moderating factors, based on the Model for Adaptation Design and Impact.

**MADI element**	**MADI response options**	**Modification of MADI response options for this study**
**Domain 1: Adaptation characteristics**		
What was modified	• Content • Contextual • Training and evaluation • Implementation and scale-up activities	Modified slightly to reflect program changes, namely replaced “training and evaluation” with “training and supportive supervision”
Nature of adaptation	Fifteen options (e.g., adding elements)	Added “increasing elements” as an option
Who participated in adaptation decision-making	• Political leaders • Program leader • Funder • Administrator • Program manager • Intervention developer/purveyor • Researcher • Treatment/intervention team • Individual practitioners • Community members • Recipients	Modified to reflect relevant roles by adding the following response options: HHC program leaders; BHP program manager; Trinity Health project manager; Trinity Health project assistant; Trinity Health hospital staff (e.g., lead physicians, nurse managers); IBCLCs, peer counselors
For whom/what was the adaptation made	• Individual • Target intervention group • Cohort/individuals that share a characteristic • Individual practitioner • Clinic/unit level • Organization • Network system/community	Modified slightly to reflect relevant levels of delivery by adding two additional response options: peer counselor and program level
**Domain 2: Possible mediating or moderating factors**		
Alignment with core functions/relationship to fidelity	• Fidelity consistent/core functions preserved • Fidelity inconsistent/core functions changed • Unknown	None
Goal/reason for adaptation	Goal: • Improve likelihood of adoption • Improve feasibility • Improve fit with recipients (appropriateness) • Address cultural factors • Increase satisfaction (acceptability) • Reduce cost • Increase reach/engagement (penetration) • Improve fidelity • Increase retention • Improve sustainability • Improve intervention effectiveness/outcomes • No goal	Added goals to reflect relevant goals, including: ensure safety, promote equity, and promote person-centeredness
	Reasons: • Sociopolitical which includes 9 response options (e.g., existing laws, political climate) • Organization/setting which includes 10 response options (e.g., available resources, competing demands)	
	• Provider which includes nine response options (e.g., race, first spoken languages, preferences, clinical judgement) • Recipient which includes 14 response options (e.g., race, access to resources, literacy, motivation/readiness)	
Systematic/unsystematic	• Systematic (i.e., made using a formal process that includes consulting data, theory, best practice, and/or stakeholders as well as considering the impact on outcomes) • Unsystematic (i.e., made without a formal process)	None
Proactive/reactive	• Proactive (i.e., made due to an anticipated obstacle) • Reactive (i.e., made due to an unanticipated obstacle)	None

MADI = Model for Adaptation Design and Impact; HHC = Hispanic Health Council; BHP = Breastfeeding Heritage and Pride program; Trinity Health = Trinity Health Of New England; IBCLCs = International Board Certified Lactation Consultants; N/A = not applicable.

#### Program meetings

Once in-depth interviews were completed, it became clear that the COVID-19 pandemic would necessitate that BHP continue virtual services for longer than anticipated. We therefore initiated a process of ongoing data collection to capture any additional adaptations made after interviews were completed, as well as the influence of these adaptations on implementation outcomes. To minimize the burden of data collection on program implementers, we collected data by attending and taking notes during bimonthly implementation team meetings and PIP meetings held between the end of October to mid-December 2020 at the three Trinity Health hospitals. We probed throughout the discussions to obtain detailed information about adaptations and their perceived impact on implementation. Meeting notes were used to add to the results from in-depth interviews. Throughout this process, we consulted with program implementers to help ensure comprehensiveness, verify interpretations, and correct categorization of adaptations.

#### Internal report on adaptations

The program manager developed a detailed report describing program adaptations made between March and July 2020 in response to the COVID-19 pandemic. To write the report, she drew on her in-depth knowledge of these adaptations given her role in the decision-making process for adaptations, managing day-to-day program operations, and providing training and supportive supervision to peer counselors throughout the pandemic. We reviewed this report to capture information for describing the elements of adaptation delineated in the FRAME.

#### Data triangulation and member checking

We integrated data from in-depth interviews, program meeting notes, and an internal report to generate detailed and comprehensive information on adaptations. After this triangulation of data was completed, we used member checking to validate the results (25). We shared a full draft of the results with four program implementers who participated in interviews to explore whether the results resonated with their experiences. After review, these program implementers shared their feedback through discussions and written notes. No major changes were recommended, though minimal additions were suggested, such as greater specificity of who was involved in making decisions to modify the program. This additional detail was then added to the results.

## Results

There were two major contextual adaptations: virtual recruitment and virtual counseling, which prompted training adaptations and content adaptations. We present the findings regarding these adaptations following the steps for retrospective application of MADI (12). First, we describe adaptations using Domain 1 (adaptation characteristics). Second, we describe relevant outcomes from Domain 3 (implementation and intervention outcomes). Third, we use constructs from Domain 2 (possible mediating or moderating factors) to help explain why and how outcomes were achieved.

### Adaptations to training and supportive supervision

#### Description of adaptations

To support all HHC staff including BHP peer counselors to plan and implement virtual services, HHC leadership provided training and support on topics such as maintaining confidentiality of client information (Table 2). Additionally, the BHP program manager immediately began providing peer counselors with training on remote work, virtual service delivery, and COVID-19 and breastfeeding (Tables 3, 4). Training on maintaining privacy and confidentiality while working remotely was emphasized because many peer counselors and clients were now at home with young children and family members, often in small spaces. Training on how to support clients during the pandemic was critical, since many clients are from communities disproportionately impacted by COVID-19 and related consequences such as job loss. This training was also essential for keeping peer counselors updated on fast-changing public health guidance regarding breastfeeding as well as rapidly evolving protocols at Trinity Health hospitals.

**Table 2:** Components of Hispanic Health Council's effort to support Breastfeeding Heritage and Pride program staff to work remotely.

**Component**	**Description**
Provision of equipment for remote work	The Hispanic Health Council (HHC) ensured that each peer counselor could connect their work cell phone and laptop to a reliable internet source at home
Support with accessing electronic systems	HHC provided support so that peer counselors could securely access the data management system and Epic
Training and support for maintaining confidentiality	HHC focused on ensuring confidentiality for all staff. Since peer counselors no longer had access to locked storage for client files at HHC and clinical sites, they were issued a mobile file cabinet that could lock and be used at home to ensure secure storage of confidential client information contained in paper forms such as visit summary forms
Transition to electronic client data	Paper forms were eventually converted to fillable PDF forms to enable electronic completion and storage of client information – a process that was well-planned and underway pre-pandemic but accelerated during the pandemic
IT support	The BHP program manager established a system for remote support to help peer counselors address technological issues that might arise

**Table 3:** Additional training provided to peer counselors during the COVID-19 pandemic.

**Training**	**Training session topics**
Working remotely	• Setting up and using Zoom and other video platforms such as WhatsApp and FaceTime • Maintaining professional appearance, deportment, and work standards • Minimizing noise and distractions in the home environment while working • Using electronic data management database
Virtual service delivery	• Maintaining privacy and confidentiality (e.g., safeguarding of client information and compliance with HIPAA requirements, with emphasis on confidentiality during video calls conducted from peer counselors' homes; ensure that the interaction could not be observed or overheard by additional persons (including children) on either side, unless permission was secured from the client) • Providing virtual breastfeeding peer counseling (e.g., asking permission before using video calls)
COVID-19 and breastfeeding	• General safety considerations during COVID-19 • Supporting women of color during the COVID-19 pandemic • Breastfeeding during the COVID-19 pandemic (e.g., most up-to-date public health guidance) • Hospital-specific inpatient/outpatient COVID-19 protocols and procedures

**Table 4:** Adaptation characteristics.

**Adaptation**	**What was modified^1^**	**Who participated in adaptation decision-making**	**Nature of content adaptation^1,2^**	**For whom/what was the content adaptation made^1,^^2^**
Working remotely	Training and supportive supervision	Hispanic Health Council (HHC) program leaders, BHP program manager, Trinity Health project manager and assistant	N/A	N/A
Virtual service delivery	Training and supportive supervision	HHC program leaders; BHP program manager	N/A	N/A
COVID-19 and breastfeeding	Training and supportive supervision	HHC program leaders, BHP program manager, Trinity Health project manager and hospital staff (e.g., lead physicians, nurse managers)	N/A	N/A
Peer counselors recruited clients virtually	Context	HHC program leaders, BHP program manager, Trinity Health project manager and assistant	N/A	N/A
Peer counselors provided counseling virtually	Context	HHC program leaders, BHP program manager, Trinity Health project manager and assistant	N/A	N/A
Increased in online educational resources	Content	BHP program manager, IBCLCs, peer counselors	Increasing elements	Program level
Provided Breastfeeding education and support in the context of COVID-19	Content	BHP program manager, IBCLCs, peer counselors, HHC program leaders, Trinity Health hospital staff	Adding elements	Program level
Expanded social and economic support	Content	BHP program manager, peer counselors, HHC program leaders	Increasing elements, adding elements	Program level

^1^Response options are based on the response options listed in MADI and modified for this study (see Table 1).

^2^According to MADI, this element is relevant for content adaptations only. Thus, for training and supportive supervision and context adaptations, we included “N/A” to denote that this element is not applicable.

Supportive supervision intensified. Weekly online group meetings were instituted so that the program manager, program IBCLC, and peer counselors could continue to share program updates, review client cases, and maintain ongoing training on relevant topics, as well as to discuss hospital-specific protocols and challenges regarding virtual service delivery and potential solutions. The program manager held individual weekly supervisory meetings with each peer counselor by phone, and the IBCLC met with each peer counselor bi-weekly or weekly over the phone to discuss clinical topics. Both the program manager and IBCLC were also available to meet with peer counselors between scheduled meetings to offer additional support.

Recognizing that some clients may not be familiar with video platforms, peer counselors were trained to assess each client's readiness to use these platforms and identify their preferred platform. Some clients did not want to use video calls, or only wanted video calls prenatally but not postpartum. Because program implementers were primarily concerned with maintaining contact at the scheduled touchpoints, peer counselors were advised to schedule video calls in place of in-person visits whenever possible, but if video calls were not possible or preferred by clients to contact them by phone and then lastly text.

#### Implementation outcomes

##### Adoption

Training and providing supportive supervision to peer counselors on setting up and using platforms like Zoom and providing virtual breastfeeding peer counseling was viewed by program implementers to be important for increasing peer counselors' use of video calls. This was particularly important given that Zoom was a new technology to many peer counselors and video calls were a different mode of service delivery that peer counselors did not readily adopt when services initially transitioned to being virtual. Training and supportive supervision also promoted the adoption of content adaptations like the provision of breastfeeding education and support in the context of COVID-19, ensuring consistency in the content delivered by all peer counselors.

##### Feasibility

Training on working remotely and virtual service delivery, as well as increased supportive supervision, enabled peer counselors to continue recruitment and service delivery during the pandemic. Specifically, program implementers emphasized that the feasibility of recruiting and delivering services virtually was largely due to peer counselors having the knowledge, skills, and ongoing assistance from program implementers to do so.

##### Appropriateness

Training influenced the appropriateness of the shift to virtual recruitment and service delivery. For example, maintaining privacy and confidentiality was viewed as an essential aspect of service delivery. Therefore, training and supportive supervision that helped ensure privacy and confidentiality was maintained made virtual recruitment and service delivery appropriate, particularly from the perspectives of program implementers.

##### Acceptability

The program manager found remote training and supportive supervision to be acceptable, in part because she was accustomed to providing some supportive supervision via phone pre-pandemic since she was based at HHC and peer counselors were based at hospitals. A dedicated time that was convenient for all peer counselors to come together for weekly meetings and receive ongoing training was viewed by the program manager as a positive consequence of the pandemic. A few peer counselors echoed this sentiment. In particular, they described the training on how to use Zoom and other platforms such as WhatsApp and FaceTime for counseling as well as the opportunity to apply this knowledge in practice as a “great learning experience.”

#### Possible mediating or moderating factors

The reason that adaptations were made to training and supportive supervision was that program implementers recognized that successful implementation of virtual recruitment, virtual service delivery, and content adaptations would require that peer counselors build on their previous training and skills. For program implementers, a primary goal for offering additional training and supportive supervision was to facilitate successful implementation of services, and to maintain service quality to the best of their ability within the constraints imposed by the COVID-19 pandemic. Because this adaptation was made due to the unanticipated challenge of the COVID-19 pandemic, it was categorized as reactive. Still, this adaptation was systematic. At the program level, program implementers with rich experience implementing BHP identified the need for this adaptation immediately once the decision was made to stop in-person service delivery. They selected the training and supportive supervision topics based on their expertise and knowledge of the specific training needs of peer counselors as well as needs identified by peer counselors themselves. At the organizational level, HHC leadership determined to provide training on topics like maintaining confidentiality based on their extensive experience implementing CHW-led programs and knowledge of what would be required for CHWs to work remotely. With a clear rationale and systematic process, the adaptations to training and supportive supervision played a key role in maintaining alignment of program services with the core function of providing high-quality breastfeeding education and lactation management services.

### Contextual adaptations

#### Virtual recruitment

##### Description of adaptation

Peer counselors identified pregnant women in Epic and received referrals from healthcare providers through Epic as well as by phone or email (Table 4). Of note is that many peer counselors started returning to hospitals in July and August of 2020, and thus, were able to resume in-person recruitment and referrals from healthcare providers.

##### Implementation outcomes

###### Feasibility

The shift to virtual recruitment was feasible in part because it occurred within the context of HHC transitioning to remote work through a well-thought-out process. Several days before BHP made the shift, senior management at HHC met to determine what would be needed for remote work, met with all staff to discuss the situation, and equipped staff including BHP peer counselors with resources and support for remote work (Table 2). Additionally, the program manager advised peer counselors to do a “trial run” to see if they could successfully work remotely, so that any issues could be addressed prior to the transition to remote work.

*What matters is the systematic approach that was taken to the behind-the-scenes stuff that aren't specific to the program model but are necessary to support it…So when we called it [work would be remote] a lot happened fast right before…to put everything in place. And after we put it all in place, there were still some things that needed fine tuning*. (HHC staff member)

Initially, recruitment of new clients through Epic was not possible because peer counselors were unable to remotely access Epic due to internet security restraints. Program implementers worked with hospital administrators to ensure proper HIPAA compliance and grant peer counselors remote Epic access. Several program implementers reported that granting peer counselors this access was a major challenge. Furthermore, although remote access was granted at the beginning of the pandemic, upgrades subsequently made to Epic for security reasons made it difficult for some peer counselors to access Epic remotely later on in the pandemic. Since many peer counselors were back onsite at hospitals when the upgrades occurred, they had less of a need to access Epic remotely. When peer counselors could access Epic, they used it to identify pregnant women to call for recruitment but quickly found that some women were reluctant to answer calls from numbers they did not recognize or were unsure why a peer counselor was calling. These “cold calls” were viewed as quite different than having a peer counselor dressed in business casual with a hospital ID meet with a woman in the prenatal clinic to discuss BHP. The program manager therefore increased referrals by regularly reminding providers that BHP was still operating. She also shared information about the program with providers who newly joined prenatal clinics and were not yet aware of BHP. She encouraged providers to refer patients to the program through Epic and to introduce the program to patients during prenatal visits so they could anticipate peer counselors' calls. Both peer counselors and program implementers emphasized the importance of referrals from providers as a determinant of feasibility with regards to recruitment both pre-pandemic and during the pandemic. Women knowing ahead of time that peer counselors would be calling made recruitment easier for peer counselors.

Most peer counselors found it more difficult to reach women by phone than in person in prenatal clinics. Pre-pandemic, providers could easily bring peer counselors in and introduce them to potential clients, which was widely considered to be a highly effective recruitment strategy. Additional perceived barriers to reaching women included women not picking up “cold calls” due to not recognizing peer counselors' phone numbers, not listening to voicemail or viewing returning voicemail messages as a “priority,” and not responding to text messages. However, peer counselors felt that it was easy to recruit women into the program once they answered their phones. As such, peer counselors perceived remote recruitment to be feasible, though it sometimes required substantial effort and persistence.

Program implementers and a couple peer counselors highlighted advantages of remote recruitment compared with in-person recruitment. For example, talking with women over the phone offered ample time to discuss BHP, whereas recruitment in hospitals felt rushed if women needed to leave soon after their prenatal visits or, in other instances, led to missed opportunities to talk with women if they left before peer counselors could catch them.

###### Appropriateness

While peer counselors thought that sharing information about the program over the phone or text messages was appropriate, they found remote communication to be less optimal for building rapport with potential clients compared with in-person communication, especially when “cold calling” women since they may wonder who the peer counselor is and why she is calling.

###### Acceptability

Peer counselors pointed out that women “love texting” about BHP, and in some cases, preferred communicating via text. In some instances, women explicitly asked peer counselors not to call them because they were working and unable to talk. Peer counselors described sharing information via text messages as more “complicated” and “tedious” than conveying information over the phone because it required them to write a lot in the text messages. However, peer counselors expressed a commitment to being responsive to women's preferences and therefore regularly communicated about BHP via text during the recruitment process.

##### Possible mediating or moderating factors

The reason BHP shifted to virtual recruitment was to adhere to an HHC policy requiring all staff to work remotely in order to follow public health guidance for social distancing, and a Trinity Health policy limiting non-hospital staff from operating within the hospital to minimize spread of SARS-CoV-2. The primary goal was to ensure the safety of program staff, peer counselors, and clients, while sustaining program operations and maintaining reach and engagement to prevent breastfeeding inequities from widening during the pandemic. The shift to conducting recruitment virtually was reactive since it was made in response to the unforeseen pandemic and systematic in that it was made with consideration of implementation and intervention outcomes. Notably, the primary driver of this adaptation was safety. Program implementers therefore made the adaptation – and made it quickly – out of an urgent need, despite knowing that doing so may have negative effects on implementation outcomes, especially in the short run. For example, program implementers and peer counselors explained that recruitment prior to and during the pandemic was generally more successful at hospitals where peer counselors had strong working relationships with providers than at hospitals where peer counselors did not work as closely with providers. However, the unexpected and immediate shift to a new referral process in response to the COVID-19 pandemic initially had a negative effect on the feasibility of recruitment, even at hospitals where peer counselors and providers worked together closely. For example, one program implementer pointed out the initial challenges faced by one hospital:

*They [peer counselors] are integrated into a great prenatal team that is so supportive that they're fluidly interacting with each other about patients all the time. And it might be that that dynamic is so rich that it's the displacement into doing it individually in a home by phone call without the clinical team all around might just be a hard adjustment to make*. (HHC staff member)

Additionally, healthcare providers who were new to prenatal clinics and thus unaware of BHP quickly learned about the program from the program manager. The process of referrals through Epic was also disrupted since gaining access to Epic while working from home took some time, further reducing the feasibility of virtual recruitment initially. Once peer counselors and providers were reminded or made aware of BHP, gained experience with the referral process, and found ways to overcome barriers to implementation like issues with Epic access, the feasibility of virtual recruitment increased. Further, once initial challenges were addressed, virtual recruitment was consistent with the core function of the program to provide integrated peer counselor-delivered services across clinical and community settings, as peer counselors continued to be an integral part of the clinical teams and communicate with providers to recruit women into the program.

### Virtual service delivery

#### Description of adaptation

Peer counselors substituted in-person visits with phone and video calls (Table 4). Of note is that some in-person counseling, while not available in the home, was offered to clients in clinic and hospital settings as peer counselors were allowed back on hospital campuses, beginning in some locations as early as July and August 2020.

#### Implementation outcomes

##### Adoption

Peer counselors initially conducted most visits through phone calls because many clients opted for phone calls over video calls. Peer counselors themselves were reluctant to use video calls. Over time, however, peer counselors adopted video calls to approximate in-person visits using each client's preferred platform. Factors that increased peer counselors' adoption of video calls included encouragement from the program manager to use video calls, individual meetings with the program manager to identify barriers to use and ways to overcome them, training on how to use video platforms, and practice with these platforms. As peer counselors' comfort with video calls grew, they began presenting video calls as a good option for counseling when speaking with clients, which in turn increased clients' uptake of counseling via video calls. To further promote adoption of video calls, HHC updated the program protocol to include video calls as a substitute for in-person visits during the COVID-19 pandemic.

##### Feasibility

Like the shift to virtual recruitment, the shift to only virtual service provision was viewed by program implementers as feasible and “smooth.” Two key facilitators were that HHC as an organization supported its various programs in transitioning to remote work and peer counselors received training and supportive supervision to deliver virtual services.

Peer counselors reported that they were typically able to reach clients, though some clients were difficult to reach. Factors that were thought to make reaching clients feasible included clients picking up calls because they recognized peer counselors' phone numbers and clients often being home during the pandemic. Communication with clients was also facilitated by healthcare providers during pregnancy and the early postpartum period but not later during the postpartum period:

*Working alongside with the clinic…a little more so that they're gather[ing] information from that mom, whether it's call them when they're there or pass them a message along from us…but that's only feasible throughout their pregnancy and early postpartum, not past 6 weeks, 'cause after that, they're not seen any more by the gynecologist*. (Peer counselor)

Perceived barriers to communication included clients frequently being too busy to talk and, in some instances, not joining scheduled video calls as a result. Additionally, some clients were unfamiliar with video platforms in the beginning. However, some peer counselors reported that clients' comfort with using video calls increased over time, hypothesizing that this was due to clients gaining experience with using video calls for counseling visits and communicating with friends and family during the pandemic. Peer counselors found reaching clients during their birth hospitalization stay and immediately after they returned home particularly challenging, as some clients did not answer their phones during this time. In addition, peer counselors viewed younger clients as harder to communicate with than older clients because many younger clients did not answer their phones or listen to voicemails, leaving texting as the only way to reach them. Texting was perceived to be feasible but less efficient than phone calls:

…*getting information from them [younger clients] takes me 3 days vs. it can take me 10 min, because they forget to text you back, and then they text you back, going with the back and forth, but I have to do it ‘cause I'd rather get somethin' than nothing. And my first go-to is a phone call, and if I call and call and call, and they're never calling me back and neither are they answering the phone, I text them*. (Peer counselor)

##### Appropriateness

Program implementers and peer counselors reported that the appropriateness of video calls as a substitute for in-person visits varied depending on the timing of the visit. Some peer counselors perceived video calls to be appropriate for delivering prenatal education because they could still show clients props such as dolls and demonstrate latching. However, HHC staff explained that part of the reason that prenatal visits are in-person is to build rapport with clients, and both HHC staff and peer counselors frequently described the challenges of building strong relationships with clients when not interacting in person. In-person visits during the postpartum period were also viewed as important for aspects of counseling like showing clients how to use a breast pump and observing a feeding to support clients with positioning and latching. To describe the value of home visits pre-pandemic, one HHC staff member highlighted that clients may feel more comfortable in their own homes than in other settings, which can facilitate the identification of challenges that may impede breastfeeding as well as potential issues that require follow-up by a healthcare provider:

*When they [peer counselor] finally got to the home, the mother felt more comfortable pulling up her shirt and pulling down her bra, and she [peer counselor] saw a serious breast anatomy problem [that she] never had seen before. So, there's things like that that happen in the comfort of one's home that may not happen elsewhere*. (HHC staff member)

The appropriateness of video calls also varied depending on the specific needs of clients. A major limitation was that peer counselors and IBCLCs were unable to offer hands-on lactation management support to assist clients with breastfeeding difficulties such as problems with latching, which was described as “best practice.” Peer counselors were also unable to observe home environments:

*The challenge is gonna be that they can't see the whole environment, they can't pick up the same way on the dynamics between people in that environment or the kinds of needs that they'd observe in that environment*. (HHC staff member)

##### Acceptability

Several peer counselors disliked that some aspects of counseling were lost when only delivering services virtually. For example, one peer counselor shared that connecting with and supporting clients in person was rewarding, but this aspect of her work was lost during the pandemic. Similarly, another peer counselor missed the in-person relationships that she had with clients, explaining that she missed being able to go to the clinic to see clients' progress and check in to see how they were doing emotionally. For one peer counselor, losing the ability to reach clients in person was stressful because she wanted to offer clients more support than what she could provide by only communicating remotely.

Peer counselors shared their views on the acceptability of virtual service delivery from clients' perspectives. Some peer counselors found phone calls to be an acceptable mode of communication for their clients, with one peer counselor noting that some clients preferred phone calls. Similarly, some peer counselors thought that clients liked video calls. For example, postpartum clients liked showing peer counselors their infants during video calls, especially when clients had built relationships with their peer counselors prenatally. At the same time, peer counselors described some challenges regarding the acceptability of virtual service delivery. For instance, one potential client decided to not participate in the program because the program was not offering hands-on support. One peer counselor shared that she did not see many clients breastfeeding during video calls, hypothesizing that clients may not want to breastfeed while cameras were on due to privacy concerns.

#### Possible mediating or moderating factors

Similar to the reasons and goals for shifting to virtual recruitment, BHP shifted to virtual service delivery to adhere to HHC and Trinity Health COVID-19 policies, with the primary goal of preventing program staff, peer counselors, and clients from being exposed to or spreading SARS-CoV-2. Maintaining service delivery also enabled BHP to sustain reach and engagement to promote breastfeeding equity during a time when health inequities were growing. By delivering virtual services, BHP was also able to retain clients in the program who may have otherwise dropped out due to not wanting to have in-person visits due to potential exposure to SARS-CoV-2 from peer counselors, especially while pregnant or having a newborn. An additional goal was to continue providing services that are person-centered, namely meeting clients' needs and preferences for the continuation of counseling during the pandemic.

Like the other adaptations, the shift to virtual service delivery was reactive since it was made in response to an unforeseen public health emergency. Program implementers reported that this adaptation was made using a systematic process that involved review of the needs of the clients and peer counselors in light of the restrictions and requirements imposed by the COVID-19 situation, and careful consideration of how to continue services in alignment with the BHP program model and protocol. This was accomplished by: drawing on the implementers' expertise on breastfeeding support in general, and the support offered by BHP specifically; close review and revision of the BHP protocol to meet emergency needs; and monitoring of program process indicators (e.g., number of clients enrolled, number of contacts including successful contacts and unsuccessful attempts and mode such as phone, text, or video call) overall and by each hospital and peer counselor.

To continuously improve implementation of virtual services, recurring program meetings and PIP meetings as well as ongoing supportive supervision of peer counselors allowed program implementers and peer counselors to identify implementation challenges and strategies for overcoming them. Thus, the adoption and feasibility of virtual service delivery continuously improved. Furthermore, HHC staff members observed that peer counselors were highly motivated to make virtual service delivery work so that they could continue to support clients:

*The people who become peer counselors are really devoted…They want to help other people. They are well trained. They are well mentored….I think that even though there are adjustments to be made, staff are really throwing themselves into this*. (HHC staff member)

With the systematic shift to virtual service delivery, BHP maintained some but not full alignment with the core function of providing high-quality breastfeeding education and lactation management services. The main departure from this core function was that best practice for breastfeeding counseling calls for some in-person support at key points, such as when women are experiencing breastfeeding challenges like problems with latching. Additionally, in-person visits are optimal for building rapport, and in-person home visits are critical for observing the home environment and identifying social determinants of health that may impede a woman's ability to meet her breastfeeding goals. This misalignment with the core function of the program was perceived to have reduced the appropriateness of services, particularly for women with breastfeeding challenges. Acceptability also decreased among peer counselors who felt that virtual service delivery resulted in a loss of some aspects of counseling like relationship building. In contrast, the shift to virtual service delivery did not lead to a misalignment with the core function of promoting breastfeeding as a social norm and an empowering practice, since peer counselors were able to continue serving as role models for clients.

### Content adaptations

#### Description of adaptations

The shift to virtual service delivery prompted three adaptations to program content, which involved increasing and adding elements (Table 4). First, to augment prenatal education delivered virtually, peer counselors shared more online educational videos and materials with clients via text messages. Second, peer counselors offered information about breastfeeding during the COVID-19 pandemic based on the latest scientific guidance. These adaptations were initiated by individual peer counselors seeking to optimize education and support for clients. Upon sharing these adaptations with the program manager, program IBCLC, and other peer counselors, a joint decision was made for all peer counselors to follow suit. Reflecting on adaptations driven by peer counselors, a program implementer shared, “Staff [including peer counselors] are unbelievable, and they're creative, and they're committed, and they're resourceful, and they're really wanting to do their jobs well. They're coming up with new ideas about how to do it well.” HHC also developed an initiative wherein two face masks were mailed to each client along with information on mask wearing and care, the importance of breastmilk for infants, and the most up-to-date guidance on infant feeding in the context of COVID-19. Peer counselors also offered emotional support to help clients cope with the stress of the pandemic: The program manager regularly reached out to maternity departments and labor and delivery units at each hospital to understand the most recent site-specific protocols and then shared this information with peer counselors. Peer counselors then shared this information with clients to help them know what to expect during the childbirth hospitalization period given hospital COVID-19 protocols such as limits on the number of support people who can be in labor and delivery and postpartum units and separation of birthing people and their infants. Third, because socioeconomic hardships increased during the pandemic, BHP expanded support for clients with social and economic needs, providing retail gift cards for purchasing essential items like diapers and food and assistance with rent and utility payments. BHP program staff also dropped off face masks, breastfeeding supplies like breast pumps, diapers, and food at clients' homes.

*One vital thing that a postpartum mother needs, prenatal, too, but particularly at this time, sometimes it's just a caring person to speak with. They [peer counselors] certainly have been there for these participants in that way. So, you know, there are the usual anxieties and concerns of pregnancy and the new baby. But with COVID-19 added on top and changes in lifestyle…They have a listening ear. You know that has been vital. It was vital before this and it continues to be even more so*. (HHC staff member)

#### Implementation outcomes

##### Feasibility

Overall, the content adaptations made to the program were perceived to be feasible. Some peer counselors noted that providing Spanish-language online educational videos and materials to Spanish-speaking clients required more effort compared with providing English-language content. For example, one peer counselor explained that there were not a lot of online videos or materials for Spanish-speaking mothers and that much of the content was misinformation, requiring her to spend time checking Spanish materials and consulting with the program IBCLC to make sure they were evidence-informed before sending them to clients. One program implementer from HHC shared that BHP was able to provide increased support to help address social and economic needs faced by BHP clients because HHC had sought and received funds for expanded support for clients of all the programs implemented by the organization.

##### Appropriateness

Content adaptations were viewed by peer counselors and program implementers as highly appropriate considering the challenges faced by clients during the COVID-19 pandemic. Furthermore, content adaptations increased the appropriateness of virtual service delivery, as they enabled peer counselors to continue to provide breastfeeding education and support and address social determinants of health.

#### Possible mediating or moderating factors

The reason for content adaptations was to address clients' limited access to resources, including information and social and economic resources. These content adaptations were made with the goal of promoting equity, delivering person-centered services, and maintaining program effectiveness. Although these adaptations were reactive, they were systematic. Program implementers planned many of these adaptations with consideration of their impact on implementation outcomes and effectiveness. When individual peer counselors made changes to the content delivered to clients given their needs and best practices, these changes were then adopted by all peer counselors.

The content adaptations were aligned with the core function of providing high-quality breastfeeding education and lactation management services. By sharing more online breastfeeding resources and offering the most up-to-date scientific guidance about breastfeeding during the pandemic, peer counselors helped to meet the needs of clients during this public health emergency, which aligns with the program's emphasis on providing services that are person-centered and evidence-informed. Moreover, addressing social determinants of health is an evidence-based strategy for supporting women in meeting their breastfeeding goals. The content adaptation to intensify this aspect of the program was therefore consistent with BHP's focus on delivering services informed by available evidence.

## Discussion

Despite the changes and challenges brought about by the COVID-19 pandemic, program implementers and peer counselors were able to shift to virtual service delivery for continued provision of breastfeeding counseling for BHP clients. A key reason for the continued delivery of services throughout the pandemic was to make sure there were no gaps in programming for clients facing breastfeeding inequities (26–30). While the shift occurred rapidly, adaptations were largely made through a systematic process. By providing peer counselors with relevant training and supportive supervision, peer counselors were largely able to continue recruitment and service delivery. Overall, virtual services worked well but were considered to be less optimal than in-person visits for several key aspects of breastfeeding counseling, such as building rapport with clients and assisting them with breastfeeding difficulties like problems with latching. Additionally, observing the home environment was not possible through virtual visits. Additions to the content delivered by the program like the incorporation of COVID-19 specific education and support were important for meeting the urgent needs of clients from communities disproportionately impacted by the COVID-19 pandemic. Since adaptations were made in response to the unanticipated COVID-19 pandemic, they were categorized as reactive. Adaptations were made for specific reasons and with clear goals like maintaining social distancing for the safety of peer counselors and clients while maintaining service delivery at a time when breastfeeding inequities could be exacerbated. Most adaptations were systematic; they were made with consideration to their impact on implementation outcomes and program effectiveness, though some negative impacts were recognized but could not be avoided since maintaining social distancing was imperative. Moreover, most adaptations were made through a well-thought-out process that considered program implementers' deep experience and best practices for peer counseling.

Some researchers have contended that reactive adaptations are more likely to be made using an unsystematic process, and an unsystematic process may compromise core functions (12). In our study, we found that changes that peer counselors were making that were both reactive and unsystematic, such as increasing online educational materials sent to clients or explaining to clients what to expect during labor and delivery given new COVID-19 protocols in hospitals, were positive and often innovative changes that allowed BHP to continue functioning during a public health emergency. As such, within the context of well-trained, experienced, and empowered CHWs who have deep knowledge of the needs and preferences of clients, adaptations at the level of CHWs coupled with regular communication to ensure consistent practices across CHWs, CHW-driven adaptations may be useful for iterative program improvement.

Our findings showed that adaptations can impact implementation outcomes in intended and unintended ways. For example, replacing home visits with video calls could increase acceptability of face-to-face interactions when peer counselors want to minimize exposure to SARS-CoV-2 but they lose rewarding aspects of their work like building relationships with and supporting clients in person. Other CHW-led programs that may be using or planning to incorporate telehealth into service delivery may benefit from considering the effect of such a change on job satisfaction and motivation among CHWs, which may influence retention. Further, these findings underscore the importance of identifying and addressing both intended and unintended impacts of adaptations on implementation outcomes (12).

We found that the fast initiation of new activities and processes to provide services virtually posed some implementation challenges, especially initially. This finding indicates that attention should not only be given to how adaptations to core functions may compromise implementation success but also how a change in forms may also affect implementation outcomes. CHW programs can undertake emergency planning so that adaptations required during emergencies are identified and planned through a systematic process that considers alignment with core functions and necessary changes to forms. Training program implementers and CHWs so that they are well-prepared to adapt programming in the face of an emergency could also enhance implementation success as programs strive to continue programming in emergency contexts.

A major strength of this study is that in addition to systematically documenting the adaptations of BHP we also evaluated the impacts of adaptations on implementation outcomes. Another strength was that multiple sources of data were used, allowing for triangulation of data. By incorporating notes from recurring program meetings into our analysis, we were able to offset the limitations of conducting in-depth interviews at one time point. Moreover, we captured the perspectives of program implementers and peer counselors who were highly involved in the adaptation process. Program implementers provided insights on adaptation characteristics such as what was modified and who participated in adaptation decision-making and potential mediators and moderators like the reasons and goals for adaptations and the extent to which the adaptations were systematic and aligned with core functions. They also described the influence of adaptations on implementation outcomes, particularly with regards to feasibility and appropriateness. Peer counselors provided insight into these topics, providing particularly rich information on what was modified and the impact of adaptations on feasibility, appropriateness, and acceptability drawing on their day-to-day experiences delivering services and interacting with clients. The use of a rapid analytic approach allowed for the timely dissemination of findings to program implementers to inform decision-making regarding the future use of telehealth.

This study has several notable limitations. First, real-time data collection on adaptations was initiated after the completion of interviews when it became clear that BHP would need to continue only delivering virtual services due to the continued COVID-19 pandemic. It is therefore possible that some adaptations that occurred prior to the initiation of real-time data collection were missed. To address this potential limitation, program implementers reviewed the final list of adaptations during the member checking process to confirm that the list was comprehensive. Second, we did not conduct interviews with healthcare providers who work closely with program implementers and peer counselors. A diversity of healthcare providers participated in PIP meetings and thus their views were captured in meeting notes and included in our analysis. Third, Kirk and colleagues point out that adaptations can have both intended and unintended impacts, and, thus, encourage researchers to consider each implementation outcome when selecting outcomes to evaluate (12). Prior to transitioning to using the MADI, we had designed our study including our in-depth interview guides to capture information on feasibility, appropriateness, and acceptability. At the same time, our qualitative research approach allowed for information regarding other implementation outcomes to emerge. Since information on adoption of virtual service delivery emerged, we reported perspectives on this additional implementation outcome. Fourth, we did not assess the impact of the shift to virtual services on the effectiveness of BHP in improving breastfeeding initiation, duration, and exclusivity. Program implementers made adaptations given the constraints of the pandemic and were aware that some adaptations were fidelity consistent while others were not and therefore program effectiveness may be negatively impacted. As such, we focused on generating information on the impact of adaptations on implementation outcomes, information of higher priority to program implementers during a public health emergency. Finally, although peer counselors were interviewed, they were not engaged in the member checking process.

Findings from this study have important implications for breastfeeding peer counselors in particular, as well as for CHWs in general. Taking our findings together, considering best practices for breastfeeding counseling, and drawing on our collective expertise in breastfeeding counseling, we do not recommend that breastfeeding counseling programs serving similar populations as BHP transition to offering only virtual services in the post-pandemic future (31). Instead, we suggest that future research studies co-design and co-evaluate a person-centered hybrid telehealth/in-person model (31–35). A hybrid model of delivery that intertwines in-person and telehealth counseling may make breastfeeding peer counseling more affordable to implement, and better meet the needs of clients who are for various reasons unable to have services provided in their homes while maintaining in-person opportunities for rapport building and hands-on lactation management support. Other CHW programs considering the incorporation of telehealth may benefit from identifying the ways in which the use of telehealth aligns (or does not align) with core functions and may influence implementation success and ultimately impact program effectiveness. It may also be advantageous for such programs to offer CHWs additional training and supportive supervision, as our study found that enhanced training and ongoing, supportive supervision was key for enabling peer counselors to deliver services virtually. This finding is consistent with existing literature and recommendations developed by the HHC in partnership with CHW policy research experts that emphasized that supportive supervision is needed for CHWs to succeed in their roles (36, 37).

Our experience conducting partner-engaged research and using a multimethod and pragmatic evaluation approach can offer lessons that can inform future CHW studies investigating program changes in both emergency and non-emergency contexts. A key lesson from our study was that strong existing partnerships between researchers and program implementers can increase readiness to immediately begin research in the context of emergencies. We also found that meaningful partner engagement substantially shaped the objectives of the research and ensured that the knowledge generated was useful for those it aimed to benefit. Clinical and community partners were largely aware of program adaptations given their role in these adaptations and frequent meetings with peer counselors and healthcare providers, and thus, the information of greatest interest and value was that of the impact of adaptations on implementation outcomes. Our multimethod approach was intentionally designed to collect the necessary data for meeting our research objectives, while reducing the burden on program implementers and peer counselors. Doing so was crucial for enhancing the feasibility and acceptability of the study given that program implementers and peer counselors were focused on maintaining service delivery during the pandemic. We added goals, such as safety and equity, to the codes available for categorizing goals for adaptations to accurately capture the rationale for BHP's shift to virtual service delivery. These additional goals may be applicable in other CHW studies, particularly those evaluating adaptations of programs designed to promote healthcare and health equity in the context of emergencies.

## Conclusion

The shift to virtual breastfeeding counseling was largely systematic and enabled service continuity for women with low incomes during the COVID-19 pandemic. This study is a case example of partner-engaged, multimethod, and pragmatic research to evaluate program adaptations in response to a public health emergency that can contribute to advancing methods for assessment of adaptations across healthcare and community settings. Our findings can help inform emergency planning and increase the speed and successful implementation of the program adaptations needed by breastfeeding peer counseling programs as well as other CHW-led programs in response to public health emergencies.

## Data availability statement

The original contributions presented in the study are included in the article/Supplementary material, further inquiries can be directed to the corresponding author.

## Ethics statement

This project was reviewed by the Yale University Institutional Review Board and exempted from requiring human subjects approval. In spite of being exempt, we chose to obtain informed consent from all study participants.

## Author contributions

ER designed the study in collaboration with all co-authors and was responsible for overseeing qualitative data collection and analysis and drafting the manuscript. MZ and NA recruited participants, conducted interviews, and contributed to data analysis. All coauthors gave substantive comments during the iterative writing of the manuscript. All authors conceptualized the study, read, and approved the final manuscript.

## Funding

ER was supported by grant number K12HL138037 from the National Heart, Lung, and Blood Institute. The content is solely the responsibility of the authors and does not necessarily represent the official views of the National Heart, Lung, and Blood Institute. This abstract was supported by the Cooperative Agreement Number 5 U48DP006380-02-00 funded by the Centers for Disease Control and Prevention, Prevention Research Center Program (PI. RP-E). Its contents are solely the responsibility of the authors and do not necessarily represent the official views of the Centers for Disease Control and Prevention or the Department of Health and Human Services. CC and RS were supported by grant number P0131174 from the W.K. Kellogg Foundation. Its contents are solely the responsibility of the authors and do not necessarily represent the official views of the W.K. Kellogg Foundation.

## Conflict of interest

The authors declare that the research was conducted in the absence of any commercial or financial relationships that could be construed as a potential conflict of interest.

## Publisher's note

All claims expressed in this article are solely those of the authors and do not necessarily represent those of their affiliated organizations, or those of the publisher, the editors and the reviewers. Any product that may be evaluated in this article, or claim that may be made by its manufacturer, is not guaranteed or endorsed by the publisher.
