# Supplementary material for: Shifting to virtual breastfeeding counseling for low-income women in the US during COVID-19: A partner-engaged multimethod evaluation of program adaptations
Source: Front Health Serv. 2022 Nov 16;2:1020326. doi: 10.3389/frhs.2022.1020326 (PMC10012814; doi:10.3389/frhs.2022.1020326)
Supplement: Supplementary file 1 [file Table_1.DOCX]

**Opening Question**

1. Before the coronavirus pandemic, what aspects of the BHP program were remote?

**TOPIC 1: Modification of BHP program**

1. What changes have been made to the BHP program so that it can operate remotely?

[ONCE WE’VE GENERATED A LIST OF CHANGES, ASK ABOUT EACH CHANGE SEPARATELY – I.E., FOR EACH CHANGE, ASK Q# 3 - 10]

1. Why was [CHANGE] made?

PROBES

- Goal
- Reasons

1. How was [CHANGE] planned for, if at all?
2. Who participated in the decision to make [CHANGE]?
3. How was the decision made to make the [CHANGE]?

PROBES

- Decision process followed

1. What helped make it possible to make [CHANGE]?
2. What challenges did you face in making [CHANGE]?
3. How did you overcome this [CHALLENGE]?

Thank you very much. You’ve shared a lot of valuable information.

1. Overall, what has helped the most in making it possible to shift from face-to-face to remote peer counseling?

PROBE

- Rapidity of change

1. Overall, what was the greatest challenge you faced in shifting from face-to-face to remote peer counseling?

PROBE

- Rapidity of change

**TOPIC 2: Implementation outcomes (feasibility, appropriateness)**

I would now like to talk about what it has been like delivering the BHP program remotely.

1. What aspects of the program have been hard to deliver remotely?

PROBE

- Why?

1. What aspects of the program have been easy to deliver remotely?

PROBE

- Why?

1. How well can peer counselors recruit mothers into the BHP program remotely?
2. How well can peer counselors support mothers remotely?

PROBES

- Different periods – prenatally, perinatally, postpartum
- Type of support – information about breastfeeding, anticipatory guidance, latch, emotional support
- Different modes – phone call, text, video chat

**Closing**

1. What aspects of the BHP program would you consider keeping remote once the coronavirus pandemic is over?

PROBES

- Why?

We have now reached the end of our discussion

1. Do you have anything else you would like to share today?

Great. Our discussion has been very helpful. I would like to thank you very much for talking with me today.
