# Supplementary material for: Shifting to virtual breastfeeding counseling for low-income women in the US during COVID-19: A partner-engaged multimethod evaluation of program adaptations
Source: Front Health Serv. 2022 Nov 16;2:1020326. doi: 10.3389/frhs.2022.1020326 (PMC10012814; doi:10.3389/frhs.2022.1020326)
Supplement: Supplementary file 2 [file Table_2.DOCX]

**Opening Questions**

1. What are some of the reasons you became a peer counselor?
2. What do you like best about being a peer counselor?

**TOPIC: Modification of BHP program**

Now I’d like to talk with you about what is has been like being a peer counselor for the BHP program during the COVID-19 pandemic.

1. What changes have you made so that you can work entirely from home?

PROBE

- Recruit mothers into the program
- Provide services – prenatal, perinatal, postpartum

1. What helped make it possible for you to make changes so that you can work entirely from home?

PROBE

- Rapidity of change
- What helped most

1. What challenges did you face in making changes so that you can work entirely from home, if any at all?

PROBE

- Rapidity of change
- Greatest challenge

1. How did you overcome these [CHALLENGES]?
2. How do you feel about the shift to everything being digital?

PROBE

- Shifting from paper forms to PDFs
- Using technology in different ways than you did before

**TOPICS: Implementation outcomes (feasibility, appropriateness, acceptability)**

1. How well can you recruit mothers into the BHP program remotely?

PROBE

- What are some of the reasons?
- Ways to make recruitment even better

I understand that peer counselors are communicating with mothers by calling and texting, video chatting, or using computers or tablets.

1. How do you communicate with mothers in the program?

PROBE

- Why?
- Points of service – recruitment, prenatally, perinatally, postpartum
- Changes over time
- Languages of mothers – English vs. non-English speakers

1. Now that you are working entirely from home, how well are you able to communicate with mothers?

PROBE

- Why?
- Reach mothers, communicate once you have reached them
- Recruitment
- Points of service – prenatally, perinatally, postpartum
- Modes of communication
- Languages of mothers – English vs. non-English speakers
- Ways to make communication even better

1. How well do you feel you can support mothers remotely?

PROBE

- Different periods – prenatally, perinatally, postpartum
- Type of support – information about breastfeeding, anticipatory guidance, latch, emotional support
- Modes of communication
- Languages of mothers – English vs. non-English speakers
- Ways to make support even better

**Closing**

Now, I’d like to talk about how your current work compares to your work before the COVID-19 pandemic. Please think back to what it was like before you were working entirely from home.

1. Overall, how has your work experience been during COVID-19 compared to before?

PROBE

- Parts of your work hardest to do remotely, easiest to do remotely

1. Which changes would you like to keep once COVID-19 is no longer an issue?

PROBE

- Why?

Great. Our discussion has been very helpful. I have asked all the questions that I have for you.

1. Do you have anything else you would like to share today?

Thank you very much for talking with me today. Your experiences and opinions are very valuable!
